# Postmarketing Surveillance of the Safety and Effectiveness of Metreleptin in Patients with Lipodystrophy in Japan

**DOI:** 10.1210/jendso/bvaf216

**Published:** 2025-12-23

**Authors:** Ken Ebihara, Tomohisa Hata, Yumi Sato, Makiko Miyano

**Affiliations:** Division of Endocrinology and Metabolism, Department of Internal Medicine, Jichi Medical University, Shimotsuke 329-0498, Japan; Pharmacovigilance Department, Shionogi & Co., Ltd., Osaka 530-0011, Japan; Medical Affairs Department, Shionogi & Co., Ltd., Osaka 530-0011, Japan; Pharmacovigilance Division, Shionogi Business Partner Co., Ltd., Osaka 541-0045, Japan

**Keywords:** HbA1c, lipodystrophy, metreleptin, safety and effectiveness, postmarketing surveillance, triglycerides

## Abstract

**Purpose:**

The long-term safety and effectiveness of metreleptin in patients with lipodystrophy were evaluated.

**Methods:**

We conducted postmarketing surveillance of all patients administered metreleptin at least once from July 2013 at its launch through July 2021 in Japan. Data were collected through July 2022. The safety analysis set included all patients with Case Report Forms (CRFs) retrieved, including those who transitioned from premarketing clinical trials and those who newly initiated metreleptin after its launch. Only the latter cases were eligible for the effectiveness analysis set. Effectiveness was evaluated by changes in glycated hemoglobin (HbA1c) and triglyceride levels.

**Results:**

CRFs were collected from 48 patients. Thirty-six were new cases, and 12 were transitioned cases. Twenty-six types of adverse drug reactions (ADRs) were reported in 15 patients. The most reported ADRs were “decreased appetite” and “neutralizing antibodies positive,” reported in 3 patients each. However, there were no safety concerns overall. Mean levels of HbA1c and triglycerides decreased at 2 or 4 months of treatment. Values thereafter remained stable for up to 7 years for HbA1c and triglycerides. Mean levels of HbA1c and triglycerides improved after 1, 2, and 3 years, although the change was not as great in partial lipodystrophy patients as in generalized lipodystrophy patients.

**Conclusion:**

Metreleptin treatment was generally well tolerated in patients with generalized and partial lipodystrophy and lowered HbA1c and triglycerides in those with generalized lipodystrophy. This surveillance demonstrated for the first time the long-term safety in the real world and the effectiveness in patients who used the medication correctly.

Lipodystrophy syndromes are a rare, potentially life-threatening, heterogeneous group of diseases that are characterized by a generalized or partial loss of body fat and a subsequent risk of severe metabolic disorders [1-3]. The prevalence of lipodystrophy has been reported to be as low as 1.3 to 4.7 cases per million individuals [4]. In Japan, the number of patients with lipodystrophy is estimated to be approximately 100 [5]. As for partial lipodystrophy, the number of reported cases is relatively small in Japan, suggesting that the undiagnosed rate of partial lipodystrophy is high.

Based on the etiology and the pattern of body fat loss, lipodystrophy syndromes can be classified into 4 major types: congenital generalized lipodystrophy, familial partial lipodystrophy, acquired generalized lipodystrophy (AGL), and acquired partial lipodystrophy [6]. Regardless of the disease type, as the loss of body fat progresses, patients often develop metabolic abnormalities such as severe insulin resistance, diabetes, hypertriglyceridemia, and nonalcoholic fatty liver disease [1, 2]. The severity of the metabolic complications is generally related to the extent of body fat loss [6]. In the past, there was no standard treatment for the metabolic complications of lipodystrophy; symptomatic therapy, dietary restrictions, and antidiabetic or antihyperlipidemic drugs were used. However, these therapies were often not effective in severe cases [7].

Leptin is an adipocyte-derived hormone that regulates energy homeostasis mainly via the hypothalamus [8, 9]. Patients with lipodystrophy exhibit low leptin levels [10]. Leptin deficiency is thought to be 1 of the main causes of metabolic complications in lipodystrophy [9]. Leptin replacement therapy with recombinant human methionyl leptin (metreleptin) has been demonstrated to improve insulin resistance, hyperglycemia, hypertriglyceridemia, and hepatic steatosis [7]. Metreleptin received its first-ever global approval in Japan in 2013 [11].

Three clinical trials of metreleptin for lipodystrophy were conducted in Japan before the approval of metreleptin. All were open-label trials, namely, an exploratory clinical trial with 11 patients (KUTR-003-0), a Japanese investigator-initiated clinical trial with 4 patients for the approval application (KUTR-003-1), and a clinical trial with 12 patients (KUTR-003-2), where the patients were recruited from the participants of KUTR-003-0 and KUTR-003-1, conducted as a Japanese Advanced Medical Trial. All these clinical trials demonstrated the efficacy and safety profiles of metreleptin. However, the number of cases in these clinical trials was very small, and long-term outcome data for metreleptin treatment in Japanese patients with lipodystrophy are lacking.

Herein, we report the results of a 9-year postmarketing surveillance (PMS) conducted in Japan. We evaluated the safety throughout the study period and the effectiveness whenever data were available, resulting in effectiveness data for up to 7 years of metreleptin use in patients with lipodystrophy. We also investigated the effectiveness of metreleptin for generalized and partial lipodystrophy. This is the first report on the safety and effectiveness of metreleptin treatment in routine clinical practice.

## Materials and Methods

### Study Design

This PMS represents observational research of all patients who were administered metreleptin at least once between July 2013 and July 2021 in Japan. Patients were registered at the initiation of metreleptin after its launch, and their clinical courses were followed over time. The administration method followed the approved dosage and regimen as described later; however, the PMS did not control the dosing schedule or dosage. This PMS was carried out in accordance with the Good Postmarketing Study Practice for medicinal products in Japan and was registered with the Japan Pharmaceutical Information Center (identifier: UMIN000051667). According to exemptions under the Good Postmarketing Study Practice Ordinance No. 171, 2004, by the Ministry of Health, Labour and Welfare, ethics approval by the institutional review board and informed consent were not required.

The approved dosage schedule included a once-daily subcutaneous injection of metreleptin at a starting dose of 0.03 mg/kg for females ages <18 years, 0.04 mg/kg for females ages ≥18 years, and 0.02 mg/kg for males. These doses could be increased over a period of about 1 month to the once-daily maintenance doses of 0.06 mg/kg for females ages <18 years, 0.08 mg/kg for females ages ≥18 years, and 0.04 mg/kg for males. Doses could be reduced as appropriate based on patient response and symptoms.

#### Study participants

The participants in this PMS comprised transitioned cases, who had continued metreleptin postmarketing after initiating treatment in the clinical trial (KUTR-003-2), and new cases who were prescribed metreleptin for the first time after its launch without prior participation in clinical trials. The safety analysis set included both transitioned and new cases for which case report forms were retrieved. The effectiveness analysis set included only the new cases adhering to the approved dosage and administration.

As a supplementary analysis, we also aggregated effectiveness data for the new cases regardless of dosage deviations.

#### Data collection

Baseline demographics, including sex, age, lipodystrophy subtype, complications of lipodystrophy, body mass index, hepatic and renal impairment status, and concomitant treatments, were collected. We set the observational time points as 2, 4, 6, and 12 months and once a year thereafter, until either treatment discontinuation or the last visit through July 2022. For each time point, we acquired the results of hematological tests and blood biochemical tests, including levels of glycated hemoglobin (HbA1c) and blood triglycerides. Blood collection was not restricted to fasting samples. Physicians were required to report when patients became pregnant or started breastfeeding. Antibody and its neutralizing activity tests were conducted by an external laboratory using the reported methods [12] upon request when antibody development was suspected due to a decreased therapeutic response or the occurrence of an adverse drug reaction (ADR).

#### Safety outcomes

Within our research group, some researchers believe that certain abnormal laboratory values, such as antibody positivity, which are not necessarily medically undesirable, should not be classified as adverse events (AEs). However, in this PMS adhering to the health authority's order, we have classified these abnormal laboratory values as AEs based on the attending physician's judgment. For example, all leptin-antibody positive and anti-leptin neutralizing antibody positive (coded in Medical Dictionary for Regulatory Activities as “antibody test positive” and “neutralizing antibodies positive,” respectively) were categorized as AEs when the attending physician reported them as AEs.

The reported terms of AEs were coded using the Medical Dictionary for Regulatory Activities, Japanese version 25.1. Both the attending physician and the sponsor physician assessed the causal relationship and the seriousness of AEs. If either physician judged that a causal relationship between the AE and metreleptin could not be clearly ruled out, the AE was defined as an ADR. Similarly, if at least 1 physician judged an AE to be serious, it was classified as a serious AE. Events regarded as serious AEs included those causing death, that were life-threatening, that required hospitalization or prolonged hospitalization for treatment, that resulted in permanent or significant disability/incapacity, that resulted in congenital abnormalities/defects in participants’ offspring, and that were judged to be medically important conditions. In the case of pregnancy or breastfeeding, information about AEs in the patient's infant was collected.

#### Effectiveness outcomes

The mean test values and percentage change from baseline levels of HbA1c and blood triglycerides at 2, 4, 6, and 12 months and once a year thereafter were evaluated for the effectiveness analysis. To examine if effectiveness differed depending on the type of lipodystrophy, HbA1c and blood triglyceride levels were tabulated in subgroups with generalized and partial lipodystrophy after 1, 2, and 3 years of metreleptin administration.

### Statistical Analysis

Summary statistics (mean and SD) were used for quantitative variables and frequencies and proportions for qualitative variables. The incidence of ADRs in the safety analysis was calculated as a safety analysis. All analyses were performed using SAS (SAS Institute Inc., SAS for Windows version 9.2 or higher).

## Results

### Demography of Participants

**Figure 1.**
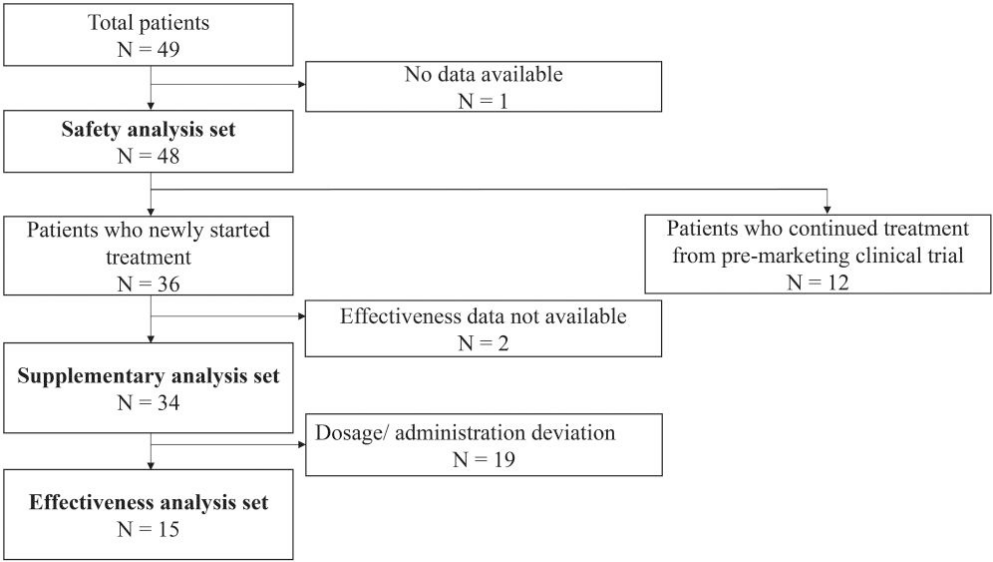
Flowchart of patient enrollment.

Forty-nine cases were enrolled in this PMS, and case report forms were collected from 48 of them; all 48 were included in the safety analysis. Out of 48 cases, 36 were new cases and 12 were transitioned cases from the premarketing clinical trials. After excluding the patients whose data were not available and those in whom dose/administration deviations were reported from the 36 new patients, 15 patients were included in the effectiveness analysis (Fig. 1). As a supplementary analysis, effectiveness was evaluated in 34 patients, including those with dose/administration deviations. The reasons for discontinuing treatment are detailed in Table S1 [13]. Seven patients discontinued the treatment due to inadequate effects, and 6 patients discontinued due to AEs.

Table 1 shows the characteristics of the analyzed subjects by analysis set. The percentage of female patients was 66.7% in the safety analysis set and 53.3% in the effectiveness analysis set. The mean age (± SD) was 27.0 ± 13.8 years for the safety analysis set and 34.3 ± 16.5 years for the effectiveness analysis set. The percentage of generalized cases of congenital generalized lipodystrophy and AGL combined was 70.8% in the safety analysis set and 60.0% in the effectiveness analysis set. The mean body mass index (± SD) was 17.2 ± 3.4 kg/m^2^ for the safety analysis set and 19.3 ± 3.6 kg/m^2^ for the effectiveness analysis set. In addition, 54.2% in the safety analysis set and 73.3% in the effectiveness analysis set took diabetic medications, 31.3% in the safety analysis set and 46.7% in the effectiveness analysis set took insulin, and 29.2% in the safety analysis set and 46.7% in the effectiveness analysis set took dyslipidemic medications at baseline.

**Table 1.** Baseline demographics

Item	Category	Safety analysis set (n = 48) n (%)/mean (SD)*^a^*	Effectiveness analysis set (n = 15) n (%)/mean (SD)*^a^*
Sex	Male	16 (33.3)	7 (46.7)
	Female	32 (66.7)	8 (53.3)
Age, years	Mean (SD)	27.0 (13.8)	34.3 (16.5)
Subtype of lipodystrophy	Generalized	34 (70.8)	9 (60.0)
	CGL*^b^*	24 (70.6)	7 (77.8)
	AGL*^b^*	8 (23.5)	2 (22.2)
	Unknown*^b^*	2 (5.9)	0 (0)
	Partial	14 (29.2)	6 (40.0)
	FPL*^c^*	4 (28.6)	1 (16.7)
	APL*^c^*	7 (50.0)	2 (33.3)
	Unknown*^c^*	3 (21.4)	3 (50.0)
BMI (kg/m^2^)	Mean (SD)	17.2 (3.4)	19.3 (3.6)
Insulin resistance	Yes	42 (87.5)	13 (86.7)
Hyperinsulinemia	Yes	33 (68.8)	9 (60.0)
Diabetes	Yes	38 (79.2)	12 (80.0)
Hypertriglyceridemia	Yes	36 (75.0)	13 (86.7)
Fatty liver	Yes	37 (77.1)	12 (80.0)
Hepatic impairment	Yes	38 (79.2)	13 (86.7)
Renal impairment	Yes	8 (16.7)	2 (13.3)
Antidiabetic agents	Yes	26 (54.2)	11 (73.3)
Insulin	Yes	15 (31.3)	7 (46.7)
Antihyperlipidemic agents	Yes	14 (29.2)	7 (46.7)

Abbreviations: AGL, acquired generalized lipodystrophy; APL, acquired partial lipodystrophy; BMI, body mass index; CGL, congenital generalized lipodystrophy; FPL, familial partial lipodystrophy.

^a^n (%) for categorical variables and mean (SD) for continuous variables.

^b ^The denominator used was the number of patients with generalized lipodystrophy in each analysis set.

^c ^The denominator used was the number of patients with partial lipodystrophy in each analysis set.

### Safety Outcomes

The mean observation period of 48 patients for the safety analysis was 4.3 years. Twenty-six different types of ADRs were reported in 15 patients (Table 2). The most reported ADRs were decreased appetite and neutralizing antibodies positive in 3 patients each.

**Table 2.** Adverse drug reactions in the safety analysis (n = 48)

Total number of cases, n	48
Patients who presented any adverse drug reaction, n	15
**Adverse drug reaction**	**Cases, n**
Anemia macrocytic	1
Anaphylactic reaction	1
Hyperglycemia	1
Hypertriglyceridemia	1
Decreased appetite	3
Acute myocardial infarction	1
Aortic valve stenosis	1
Hypertension	1
Pleural effusion	1
Diarrhea	1
Hepatic function abnormal	1
Eczema	1
Pruritus	1
Injection site erythema	2
Injection site pruritus	1
Therapeutic response decreased	1
Injection site swelling	1
Blood immunoglobulin E increased	1
Eosinophil count increased	1
Glucose tolerance decreased	1
Glycosylated hemoglobin increased	1
Vitamin B12 decreased	1
Weight decreased	2
Antibody test positive	2
Neutralizing antibodies positive	3
Leptin level increased	1

Serious ADRs were antibody test positive, neutralizing antibodies positive, anaphylactic reaction, acute myocardial infarction, aortic valve stenosis, pleural effusion, and eosinophil count increased, reported in 1 patient each. Two patients who developed serious ADRs (acute myocardial infarction, pleural effusion) died during or after discontinuation of the treatment. The associations between the ADRs in those patients and metreleptin treatment were not clear. ADRs that caused the discontinuation of metreleptin treatment included anaphylactic reaction, eosinophil count increased, and blood immunoglobulin E increased. An anaphylactic reaction was observed in 1 patient. The patient exhibited a possible anaphylactic reaction about 2 months after starting metreleptin treatment. The ADR resolved the day after discontinuation of metreleptin. The same patient resumed metreleptin treatment after a 10-day interval, and again, an anaphylaxis-like reaction occurred about 1½ months after restarting. The patient recovered from the reaction the day after discontinuation of metreleptin and the administration of adrenaline. “Eosinophil count increased” and “blood immunoglobulin E increased” were reported simultaneously in another patient, who also presented with eczema and pruritus.

One patient became pregnant, delivered, and breastfed her baby during leptin treatment. No ADRs were reported in either the patient or her baby.

### Effectiveness Outcomes

**Figure 2.**
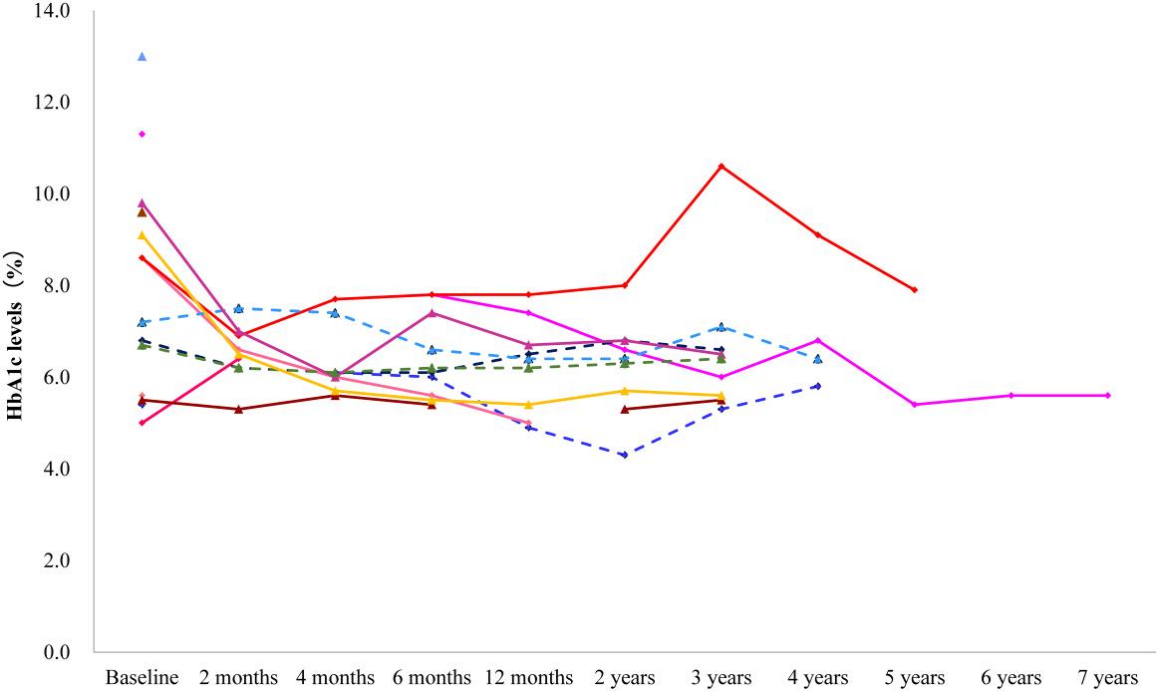
Individual patient changes in glycated hemoglobin levels over time in the effectiveness analysis (n = 14). Each line represents individual patient data. Solid line represents generalized lipodystrophy patients and dashed line represents partial lipodystrophy patients. Patients with no data during the period listed on the x-axis (effectiveness analysis timing) were excluded.

Changes in HbA1c levels over time for individual patients are shown in Fig. 2. In most patients, HbA1c levels decreased by the second month and remained stable for up to the seventh year thereafter. The mean changes from baseline in effectiveness outcomes are shown in Table 3. Mean HbA1c levels (percent) decreased from a baseline of 7.90 to 6.44 at the fourth month of treatment. This reduced level was maintained through the seventh year. In the supplementary analysis, the mean HbA1c and triglyceride values at each time point were also calculated for a population including patients in whom dose/administration deviations were reported. In this analysis, the total number of patients with either or both HbA1c and triglyceride values was 34 (28 for HbA1c and 31 for triglyceride). The results for the supplementary analysis were similar to those for the effectiveness analysis (Table S2) [13]. The reduction in HbA1c was observed at 4 months of treatment, and the decreased level was maintained until the eighth year.

**Table 3.** Mean changes from baseline in effectiveness outcomes (effectiveness analysis)

	Change from baseline to time point										
Item	2 months	4 months	6 months	12 months	2 years	3 years	4 years	5 years	6 years	7 years	8 years
HbA1c*^a^*											
Number of patients*^b^*	9	10	10	9	9	9	4	2	1	1	0
Mean value at baseline for the patients with available data, %	7.48	7.90	7.90	8.17	7.82	7.82	8.13	9.95	11.30	11.30	—
Mean value after administration at each time point, %	6.51	6.44	6.44	6.26	6.24	6.62	7.03	6.65	5.60	5.60	—
Percentage change from baseline, %*^c^*	−9.8	−15.0	−15.6	−21.1	−17.5	−11.8	−9.4	−30.2	−50.4	−50.4	—
Blood triglycerides											
Number of patients*^b^*	11	13	9	10	8	9	4	2	1	1	0
Mean value before administration, mg/dL	380.1	405.4	471.9	450.3	514.3	485.6	434.8	330.0	513.0	513.0	—
Mean value after administration, mg/dL	288.8	283.7	264.6	262.6	245.0	294.0	182.0	190.0	131.0	193.0	—
Percentage change from baseline, %*^c^*	10.4	−9.6	−23.6	−32.6	−13.8	−16.5	−36.1	−33.6	−74.5	−62.4	—

Abbreviations: HbA1c, glycated hemoglobin.

^a^HbA1c was converted to National Glycohemoglobin Standardization Program value.

^b^Patients receiving continuous administration at each time point and whose HbA1c or blood triglyceride level was measured.

^c^Average value of “(test value for each period–value before start of administration)/value before start of administration” for each case.

**Figure 3.**
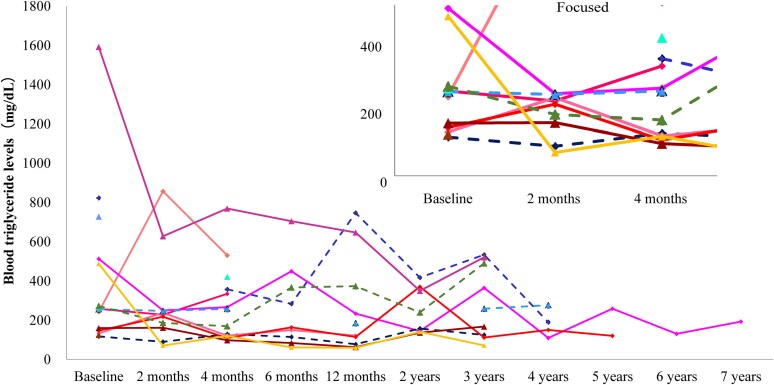
Individual patient changes in blood triglyceride levels over time in the effectiveness analysis (n = 15). Each line represents individual patient data. Solid line represents generalized lipodystrophy patients and dashed line represents partial lipodystrophy patients. Patients with no data during the period listed on the x-axis (effectiveness analysis timing) were excluded.

Triglyceride levels decreased in the majority of patients in the second month (Fig. 3), although the mean value did not show a decrease (Table 3). Furthermore, most of them remained stable for up to the seventh year, although there were some fluctuations and only 1 patient was included after the sixth year. The mean changes from baseline showed that triglyceride levels began to decrease in the fourth month, and reduced levels were observed at all time points from the fourth month to the twelfth month, with a maximum reduction of 32.6% (Table 3). After that, a similar or greater decrease was observed for up to the seventh year. In the analysis of 34 patients, triglyceride levels decreased at the fourth and twelfth month, and a similar or greater decrease was observed for up to the seventh year (Table S2) [13].

In another analysis, changes in HbA1c and triglyceride levels at the first, second, and third years were calculated for generalized and partial lipodystrophy (Table 4). The baseline values for patients whose data were available at each time point were presented alongside the mean values at each corresponding time point (Table 4). Table 4 shows the mean HbA1c and triglyceride levels at 1, 2, and 3 years after the initiation of metreleptin. As this study is observational, not all patients’ data were available for all time points; therefore, the mean scores for each time point were not derived from the exact same cohort. The mean HbA1c levels at baseline for the cohorts at years 1, 2, and 3 were 9.48%, 8.86%, and 8.86%, respectively. After metreleptin treatment, the mean HbA1c levels were 6.46% for year 1, 6.48% for year 2, and 6.84% for year 3. In partial lipodystrophy, the mean HbA1c levels for the cohorts at years 1, 2, and 3 were all 6.53%. After metreleptin treatment, the mean HbA1c levels were 6.00% for year 1, 5.95% for year 2, and 6.35% for year 3, indicating that the HbA1c levels were well controlled under 7%. The mean triglyceride level in generalized lipodystrophy decreased from 505.5, 580.0, and 580.0 mg/dL to below 250 mg/dL at the first, second, and third year, respectively, from the start of metreleptin treatment. Although the mean triglyceride level in partial lipodystrophy decreased from 404.7 mg/dL to 272.0 mg/dL at the second year, it was around 350 mg/dL and almost the same before and after treatment at the first and third years. The HbA1c and triglyceride levels were calculated for generalized and partial lipodystrophy, also using the data set of the 34 patients, including those with dosage/administration deviations (Table S3) [13]. The mean HbA1c level in generalized lipodystrophy decreased from 7.84% to 8.13% and in partial lipodystrophy from 7.39% to 7.45% to less than 7%, with a similar degree of improvement at the first, second, and third year. The mean triglyceride level in generalized lipodystrophy decreased from around 1000 mg/dL and in partial lipodystrophy from around 500 mg/dL to less than 300 mg/dL, and the degree of improvement was greater in generalized than in partial lipodystrophy at all time points.

**Table 4.** Effect of lipodystrophy type and diabetes on effectiveness outcomes (effectiveness analysis)

	Category	Number of patients*^a^*	Mean before administration	Mean after administration	Percentage change from baseline (%)*^b^*
Effect of lipodystrophy type on reduction in HbA1c levels over time*^c^*					
After 1 year	Generalized	5	9.48	6.46	−31.6
	Partial	4	6.53	6.00	−8.1
After 2 years	Generalized	5	8.86	6.48	−24.0
	Partial	4	6.53	5.95	−9.4
After 3 years	Generalized	5	8.86	6.84	−19.2
	Partial	4	6.53	6.35	−2.7
Effect of lipodystrophy type on reduction in blood triglyceride levels over time					
After 1 year	Generalized	6	505.5	206.8	−48.7
	Partial	4	367.5	346.3	−8.4
After 2 years	Generalized	5	580.0	228.8	−16.6
	Partial	3	404.7	272.0	−9.1
After 3 years	Generalized	5	580.0	247.6	−40.0
	Partial	4	367.5	352.0	12.9

Abbreviations: HbA1c, glycated hemoglobin.

^a^Patients receiving continuous administration at each time point and whose HbA1c or blood triglyceride level was measured.

^b^Average value of “(test value for each period–value before start of administration)/value before start of administration” for each case.

^c^HbA1c was converted to National Glycohemoglobin Standardization Program value.

## Discussion

Although several clinical trials have been conducted since the first clinical trial with metreleptin in patients with lipodystrophy was reported in 2002 [7, 14, 15], the number of patients in those trials was small, and safety data were insufficient. Furthermore, there are no reports so far of real-world clinical practice surveys that are not clinical trials. We conducted this PMS on all the patients treated with metreleptin after its launch. This is the first real-world survey of metreleptin treatment for lipodystrophy with a relatively large number of patients (48 cases) and a long observation period (up to 9 years).

In Japan, metreleptin treatment for patients with lipodystrophy began as a clinical study at Kyoto University in 2002. The investigator-initiated trial KUTR-003-1 was conducted on 4 new cases from 2010 to 2011 for regulatory approval. In addition, another clinical trial under the evaluation system of highly medical technology (KUTR-003-2) was conducted, in which a total of 12 patients from the clinical study and the investigator-initiated trial were treated with metreleptin. In KUTR-003-1, the most reported ADRs were headache, alopecia, and skin dryness, each observed in 2 patients. In KUTR-003-2, several ADRs were also reported; however, most of these could not be predicted from the known physiological effects of leptin. On the other hand, in our PMS, with more patients and a long observation period, 5 ADRs reported in more than 2 patients (decreased appetite, weight decreased, injection site erythema, neutralizing antibodies positive, and antibody test positive) could be caused by the physiological effects of leptin or immune-related reactions; thus we should continue to be aware of these ADRs.

In clinical trials of metreleptin conducted in the United States and Europe, acute pancreatitis after sudden discontinuation of metreleptin in patients with a history of pancreatitis and hypertriglyceridemia was reported [16, 17]. Hypoglycemia is a relatively frequent ADR, occurring in 26.1% of patients in 1 report [18]. However, neither was observed during this PMS. The frequency of hypoglycemia accompanied by metreleptin treatment may vary with concomitant medications, including insulin.

After 3 patients with AGL and severe blood abnormalities were reported to have developed T-cell lymphoma during metreleptin treatment [19], the Food and Drug Administration issued a warning of the risk of T-cell lymphoma in patients with AGL when approving metreleptin (MYALEPT (metreleptin) prescribing information. Cambridge, MA: Aegerion Pharmaceuticals, Inc; 2015) [20]. However, since then, the development of T-cell lymphoma has been reported in patients with AGL without metreleptin treatment, suggesting that metreleptin treatment may not be related to the development of T-cell lymphoma [19]. These reports could suggest that patients with AGL may frequently have concomitant T-cell lymphoma because AGL and T-cell lymphoma have a common pathogenetic basis via an autoimmune mechanism [19]. In this PMS, no patient was reported to develop T-cell lymphoma during metreleptin treatment, but one of the enrolled patients with AGL had a history of malignant lymphoma (T-cell lymphoma).

Given the high incidence of anti-leptin antibodies with metreleptin treatment [12], we should be aware of immune-related reactions to metreleptin when treating with metreleptin. In this PMS, 7 different ADRs were judged to be serious and were reported in one patient each. Of these, 2 immune-related ADRs led to the discontinuation of metreleptin treatment. Each attending physician judged “anaphylactic reaction” clearly related to metreleptin treatment and “eosinophil count increased” probably related to metreleptin. Both ADRs can be allergic reactions to metreleptin.

Two patients died during or after the metreleptin treatment; however, the associations between the ADR causing death and metreleptin treatment were not clear. One patient, who died from “acute myocardial infarction,” had a history of myocardial infarction 3 times before initiating metreleptin treatment. Therefore, this fourth acute myocardial infarction could be attributed to the patient's preexisting condition. “Pleural effusion” was classified as an ADR, because the cause of “pleural effusion” was unknown, and the AE was classified as an ADR when the association with metreleptin could not be definitively ruled out.

In addition to the T-cell lymphoma, the FDA issued a warning of the risk of the development of antibodies with neutralizing activity resulting in increased risk of infection or worsening of metabolic control when approving leptin [MYALEPT (metreleptin) prescribing information; Aegerion Pharmaceuticals, Inc., Cambridge, MA, 2015]. In the analysis of the immunogenicity of metreleptin across the clinical development program, with a total of 579 metreleptin-treated patients with obesity and 134 metreleptin-treated patients with lipodystrophy, anti-leptin antibodies developed in most patients (obese: 96-100%; lipodystrophy: 86-92%) [12]. In this analysis, 3 patients with obesity developed in vitro neutralizing activity coincident with weight gain and 4 patients with generalized lipodystrophy developed in vitro neutralizing activity concurrent with worsened metabolic control. In this PMS, “neutralizing antibodies positive” was reported in 3 patients. In all 3 patients, physicians suspected the development of anti-leptin neutralizing antibodies due to worsening of the patients’ symptoms during metreleptin treatment. While anti-leptin neutralizing antibody testing was not performed in one patient, the other 2 were tested and were judged as positive. Because no systematic testing was conducted in this PMS, the specific frequencies of antibody development and anti-leptin neutralizing antibody development in Japanese patients are still unknown. However, this surveillance revealed that anti-leptin neutralizing antibodies can develop in Japanese patients treated with metreleptin.

The effectiveness of metreleptin treatment was evaluated by the change in HbA1c and triglyceride levels. Consistent with previous results from clinical trials in Japan and other countries [7, 14, 15, 21], both values decreased early, 2 or 4 months after starting metreleptin treatment in this PMS, although the degree of change differed between generalized and partial lipodystrophy. The data presented here are not from clinical trials but only from postmarketing practice. Thus, the results of this PMS demonstrate that metreleptin treatment in clinical practice improves hyperglycemia and hypertriglyceridemia in patients with generalized lipodystrophy and may help achieve good glycemic and lipid control in those with partial lipodystrophy. Considering the low baseline values in patients with partial lipodystrophy, we believe metreleptin treatment may still provide therapeutic benefit in patients with partial lipodystrophy. Further research is warranted to confirm the effectiveness of metreleptin in patients with partial lipodystrophy, either through studies with larger sample sizes or those including patients with more advanced disease. This is the first report of metreleptin treatment in patients with lipodystrophy to analyze only postmarketing data that does not include clinical trial data.

Excluding case reports, the longest effectiveness evaluation periods for metreleptin treatment in patients with lipodystrophy to date in reported clinical trials are 4 years for generalized lipodystrophy and 3 years for partial lipodystrophy [16, 22]. In this PMS, data for HbA1c and triglyceride were collected for 7 years, combining generalized and partial lipodystrophy. The proportion of patients with generalized lipodystrophy among those analyzed for effectiveness was 60%. Considering differences in the degree of therapeutic effect but also in the long-term stability of this effect between generalized and partial lipodystrophy [16, 22, 23], we should evaluate generalized and partial lipodystrophy separately.

In a report from France that combined clinical trials and postmarketing data from 47 patients, both mean HbA1c and fasting triglyceride levels in generalized lipodystrophy improved early with metreleptin treatment, and the improvement was stable for over 15 months [23]. However, in the patients with partial lipodystrophy of this report, the mean HbA1c level did not improve throughout the treatment period, and the mean fasting triglyceride level decreased once early with treatment but then worsened from pretreatment levels. A report of clinical trials from the US National Institutes of Health also showed both mean HbA1c and fasting triglyceride levels in generalized lipodystrophy improved early after the start of metreleptin treatment, and the improvement was stable for up to 4 years [22]. In partial lipodystrophy of this report, although the improvement was smaller than in generalized lipodystrophy, the mean HbA1c level improved early with metreleptin treatment, and the improvement was stable for up to 3 years [16]. However, the mean triglyceride level in partial lipodystrophy gradually worsened after early improvement and became higher than pretreatment levels by the third year. For generalized lipodystrophy, both the degree of therapeutic effect on and long-term stability of HbA1c and triglyceride levels were almost the same between this PMS and previous reports from France and the United States [22, 23]. In contrast, for partial lipodystrophy, both the degree of therapeutic effect and its stability varied from report to report. Consistently, in our survey, the degree of improvement in both HbA1c and triglyceride levels was greater in generalized lipodystrophy than in partial lipodystrophy at the first, second, and third year from the start of metreleptin treatment. However, the mean levels before metreleptin treatment are much lower in partial lipodystrophy patients than in generalized lipodystrophy patients, and the mean levels of HbA1c and triglyceride after metreleptin treatment did not differ greatly between generalized and partial lipodystrophy except for the first year, suggesting that the difference in the percentage change from baseline with metreleptin treatment between generalized and partial lipodystrophies depends on their pretreatment levels. In addition, lifestyle and other background factors may have a relatively greater influence on the development of metabolic disorders in partial lipodystrophy than in generalized lipodystrophy.

In this PMS, dosage/administration deviations were identified in 21 of the 36 new cases. The reasons for these deviations can be attributed to the complexity of drug dissolution and the complexity of calculating the amount of drug solution according to sex, age, and body weight. Supplementarily, the effectiveness analysis was also performed with 34 cases, including 19 patients whose data were available out of 21 patients with dosage and administration deviations. The consistent results of the effectiveness analysis including the patients with deviations would support the real-world effectiveness of metreleptin.

This PMS has some limitations. First, the number of patients included is limited because lipodystrophy is a rare disease. Second, because this is an observational study, concomitant medications such as those for diabetes and dyslipidemia are not controlled. Moreover, the values of HbA1c or triglyceride are missing for some patients and time points, with some patients dropping out due to inadequate effects; this may cause bias in the effectiveness analysis. Further, the values of triglyceride fluctuate in some patients as the data can include ad libitum triglyceride evaluated in routine clinical settings, not restricted to fasting samples. In our study, we were unable to evaluate the relationship between blood leptin level and the effectiveness of metreleptin treatment, because data for blood leptin levels were not necessarily collected in this survey.

There were no safety concerns requiring additional safety measures, which refer to regulatory actions such as revising the package insert. This indicates that metreleptin treatment was generally well tolerated in our study. However, metreleptin treatment was discontinued in 2 patients due to ADRs that may have been allergic reactions to metreleptin. Therefore, continued attention should be paid to ADRs caused by allergic reactions when treating with metreleptin. The effectiveness analysis of this study suggested that metreleptin is effective against lipodystrophy in real-world clinical practice when used correctly, consistent with the results of previously reported clinical trials. Although the effectiveness cannot be simply compared with generalized lipodystrophy due to the different metabolic severity, at least the HbA1c and triglyceride values were stable during leptin treatment in partial lipodystrophy. Since the long-term prognosis of metreleptin is still unknown, continued careful observations are needed.

## Data Availability

Restrictions apply to the availability of some or all of the data generated or analyzed during this study to preserve patient confidentiality or because they were used under license. The corresponding author will on request detail the restrictions and any conditions under which access to some data may be provided.

## Clinical Trial Information

UMIN000051667.
